# Polygenic risk score penetrance & recurrence risk in familial Alzheimer disease

**DOI:** 10.1002/acn3.51757

**Published:** 2023-03-22

**Authors:** Min Qiao, Annie J. Lee, Dolly Reyes‐Dumeyer, Giuseppe Tosto, Kelley Faber, Alison Goate, Alan Renton, Michael Chao, Brad Boeve, Carlos Cruchaga, Margaret Pericak‐Vance, Jonathan L. Haines, Roger Rosenberg, Debby Tsuang, Robert A. Sweet, David A. Bennett, Robert S. Wilson, Tatiana Foroud, Richard Mayeux, Badri N. Vardarajan

**Affiliations:** ^1^ Department of Neurology, Taub Institute for Research on Alzheimer's Disease and the Aging Brain and the Gertrude H. Sergievsky Center Columbia University and the New York Presbyterian Hospital New York New York USA; ^2^ Department of Medical and Molecular Genetics, National Centralized Repository for Alzheimer's Disease and Related Dementias (NCRAD) Indiana University School of Medicine Indianapolis Indiana USA; ^3^ Department of Genetics & Genomic Sciences, Ronald M. Loeb Center for Alzheimer's disease Icahn School of Medicine at Mount Sinai New York New York USA; ^4^ Department of Neurology, Mayo Clinic Rochester Minnesota USA; ^5^ Department of Psychiatry Washington University in St. Louis St. Louis Missouri USA; ^6^ John P Hussman Institute for Human Genomics, Dr. John T Macdonald Foundation Department of Human Genetics University of Miami Miller School of Medicine Miami Florida USA; ^7^ Department of Population & Quantitative Health Sciences and Cleveland Institute for Computational Biology Case Western Reserve University Cleveland Ohio USA; ^8^ Department of Neurology University of Texas Southwestern Medical Center at Dallas Dallas Texas USA; ^9^ GRECC VA Puget Sound, Department of Psychiatry and Behavioral Sciences University of Washington Seattle WA USA; ^10^ Departments of Psychiatry and Neurology University of Pittsburgh Pittsburgh Pennsylvania USA; ^11^ Rush Alzheimer's Disease Center Rush University Medical Center Chicago Illinois USA

## Abstract

**Objective:**

To compute penetrance and recurrence risk using a genome‐wide PRS (including and excluding the *APOE* region) in families with Alzheimer's disease.

**Methods:**

Genotypes from the National Institute on Aging Late‐Onset Alzheimer's Disease Family‐Based Study and a study of familial Alzheimer's disease in Caribbean Hispanics were used to compute PRS with and without variants in the 2 MB region flanking *APOE*. PRS was calculated in using clumping/thresholding and Bayesian methods and was assessed for association with Alzheimer's disease and age at onset. Penetrance and recurrence risk for carriers in highest and lowest PRS quintiles were compared separately within *APOE‐ε4* carriers and non‐carriers.

**Results:**

PRS excluding the *APOE* region was strongly associated with clinical and neuropathological diagnosis of AD. PRS association with AD was similar in participants who did not carry an *APOE‐ε4* allele (OR = 1.74 [1.53–1.91]) compared with *APOE*‐ε4 carriers (1.53 [1.4–1.68]). Compared to the lowest quintile, the highest PRS quintile had a 10% higher penetrance at age 70 (*p* = 0.0006) and a 20% higher penetrance at age 80 (*p* < 10e‐05). Stratifying by *APOE‐ε4* allele, PRS in the highest quintile was significantly more penetrant than the lowest quintile, both, within *APOE‐ε4* carriers (14.5% higher at age 80, *p* = 0.002) and non‐carriers (26% higher at 80, *p* < 10e‐05). Recurrence risk for siblings conferred by a co‐sibling in the highest PRS quintile increased from 4% between the ages of 65–74 years to 39% at age 85 and older.

**Interpretation:**

PRS can be used to estimate penetrance and recurrence risk in familial Alzheimer's disease among carriers and non‐carries of *APOE‐ε4.*

## Introduction

Alzheimer's disease can result from rare, single mutations, but nearly all other forms are polygenic. Indeed, the largest genome‐wide array to date identified 38 genetic loci associated with late onset Alzheimer's disease,^1^ confirming its polygenic nature. Only a fraction of the heritability of Alzheimer's disease has been defined and the search for additional genetic variants continues. This reality complicates genetic counseling and discussions of recurrence risk in multiply affected families. Even the absence of the *APOE‐ε4* allele, the most robust genetic risk factor, does not ensure escaping the disease.

The incorporation of polygenic risk scores (PRS) to assess genetic risk for disease has been used, primarily among unrelated individuals. However, PRS has been helpful in assessing risk among families with other types of inherited disease such as breast cancer.^2^, ^3^, ^4^ The PRS is a useful and inexpensive method to determine penetrance and recurrence risk before onset of Alzheimer's disease in families. Previously, genome‐wide risk variants PRS (excluding the *APOE* region) explained 7% of the phenotype variance and 24% when genotypes containing the *APOE‐ε4* and ‐*ε2* alleles were included.^5^

The assessment of penetrance and recurrence risk in families with Alzheimer's disease using a PRS based on genome wide array data^1^ and *APOE* genotyping was generated in the National Institute on Aging Late‐Onset Alzheimer's Disease Family‐Based Study (NIA‐LOAD FBS) and repeated in a group of Caribbean Hispanics families. Penetrance, or the extent to which a genetic variant or set of variants were observed in individuals with Alzheimer's disease and recurrence risk, or the probability of an inherited disorder such as Alzheimer's disease in one family member will occur again in other family members were estimated using PRS and compared with similar analyses using *APOE‐ε4* allele alone. To assess the potential clinical utility of PRS in addition to *APOE‐ε4* allele, we compared the penetrance of highest and lowest quintile PRS independently in *APOE*‐ε4 carriers and non‐carriers. We chose the highest and lowest PRS quintiles of the distribution for comparison with *APOE‐ε4* allele because the percentage of individuals in each quintile is similar to the frequency of *APOE‐ε4* carriers in the population.

## Methods

### NIA‐LOAD family‐based study

This study recruited multiplex families across the United States. Families were included if at least one member had a diagnosis of definite or probable Alzheimer's disease^6^, ^7^ with onset after age 60 and a sibling with definite, probable, or possible disease with a similar age at onset. For the analyses described here, we obtained genome‐wide single nucleotide polymorphisms (SNPs) in 3676 individuals, including 1748 (47.5%) affected and 1928 (52.5%) unaffected participants over 65 years old (Table 1). Of these 2830 individuals belonged to 666 families averaging four individuals per family. Data from 846 individuals who met clinical criteria for Alzheimer's disease but did not have an affected family member were also included in these analyses based on their family history. We elected to include these individuals because they represented the types of families typically encountered in clinical practice.

**Table 1:** Demographics of NIA‐LOAD FBS and EFIGA cohort.

	NIALOAD
Total samples	3676
Number of cases	1748
Number of controls	1928
Number of unrelated individuals	846
Number of families	666
Mean number of individuals per family	4.25 ± 2.67
Average age of cases	74.66 ± 7.87
Average age of controls	77.37 ± 7.95

	EFIGA		
	Full dataset	Training	Testing
Total samples	8512	5110	3402
Number of cases	3317	1986	1331
Number of controls	5195	3124	2071
Number of unrelated individuals	6474	4011	2751
Number of families^1^	464 (100%)	307 (66%)	211
			−45%
Mean number of individuals per family	4.39	3.58	3.09
Average age of cases	76.53	76.42	76.68
Average age of controls	75.5	75.55	75.42

To estimate genome‐wide effect‐sizes of variants in a Caribbean Hispanic population, we randomly divided the full dataset into training and testing sets (preserving the ratio of affected and unaffected individuals). Variant effect‐size estimates for AD‐association were computed in the training dataset across the genome. PRS was computed in the testing dataset using the effect sizes from the training dataset. Penetrance and recurrence risk of the PRS was subsequently computed in the testing dataset.

^1^ Individuals were randomly split between training and testing datasets, which resulted in some families having members in both training and testing datasets.

Demographic information, diagnosis, age at onset for patients with Alzheimer's disease, method of diagnosis, Clinical Dementia Rating Scale,^8^ and the presence of other relevant health problems was available for each individual. The age at onset for patients was the age at which the family first observed signs of impaired cognition. For unaffected family members, we used their age at the time of their latest examination without impairment. Each recruitment site used standard research criteria for the diagnosis of Alzheimer's disease.^7^

For deceased family members who had undergone autopsy, the results were used to determine the diagnosis. In total, neuropathological confirmation of Alzheimer's disease was available for 371 individuals (183 from 76 families, and 188 unrelated AD patients), of 352 were from participants of European ancestry. For analyses, clinical Alzheimer's disease was defined as any individual meeting NINCDS‐ADRDA criteria for probable or possible Alzheimer's disease^7^ and definite Alzheimer's disease when CERAD pathological criteria^9^ was met postmortem.

### Caribbean Hispanic families (EFIGA)

To validate the results observed in NHW families, we used families from a different ethnic group to investigate our approach to penetrance and recurrence risk. We computed the penetrance and recurrence risk in 211 Caribbean Hispanic families using data from a group of families from the Dominican Republic, Puerto Rico, and New York (Table 1). Recruitment, study design, adjudication, and clinical assessment of this cohort was previously described^10^ as were details of genome‐wide SNP data, quality control and imputation procedures of the GWAS data.^11^, ^12^ Participants were followed every 2years and evaluated using a neuropsychological battery,^13^ a structured medical and neurological examination and an assessment of depression.^14^, ^15^ The Clinical Dementia Rating Scale (CDR)^16^, ^17^ and functional status were done and the clinical diagnosis of Alzheimer's disease was based on the NINCDS‐ADRDA criteria.^18^, ^19^ There were no publicly available genome‐wide association studies in Caribbean Hispanics, thus we conducted a genome‐wide association study in 1099 individuals from 307 families and 4011 unrelated cases and controls to estimate Alzheimer's disease association effect‐sizes for common variants. We subsequently computed PRS in the 211 families (651 individuals) and 2751 unrelated cases and controls and estimated penetrance and recurrence risk.

### PRS (without *APOE* region) and PRS.AD

To evaluate the utility of PRS and to compare its use with *APOE‐ε4* genotype, we constructed the PRS excluding SNPs in the 2 MB region flanking the *APOE* region (human genome b38, chromosome 19: 42,905,791–46,909,393). To estimate the additive effect of *APOE* on PRS, we computed PRS.AD defined as sum of PRS without *APOE* and the two SNPs that code for the *APOE* ε4 allele (rs429358) and *APOE* ε2 allele (rs7412). It has been previously shown^5^ that adding only the *APOE* ε2 and ε4 SNPs to the PRS and excluding the *APOE* region provides better performance than including the entire *APOE* region.

### PRS estimation in the NIA‐LOAD FBS families using PRSice

PRS was estimated using PRSice^20^, ^21^ using genome‐wide SNPs excluding the *APOE* region and standard parameters. Genome‐wide common SNPs (MAF≥0.01) with were auto‐clumped for LD (*p*‐value threshold = 0.1, *r* ^2^ < 0.2). PRS was adjusted for the first three principal components to account for population substructure. To determine weights for the PRS in the NIA‐LOAD FBS we calculated using effect size estimates from a large meta‐analysis study (base dataset) across multiple consortia.^22^ The meta‐analysis study included 71,880 individuals clinically diagnosed with AD or designated AD by proxy and 383,378 controls. A portion of NIA‐LOAD families were included in the meta‐analysis study, representing only 0.054% of the total dataset (1.77% of the cases and 0.03% of controls). Due of this minimal overlap, and heterogenous datasets in the meta‐analysis study, we derived robust estimates of PRS. For sensitivity analysis, we also computed the PRS only in non‐overlapping families with the base dataset and found the estimates were almost identical to those using both overlapping and non‐overlapping families. Thus, we report results from the full list of families in this manuscript. The PRS in the NIA‐LOAD FBS included only participants with genetically determined European ancestry. The details of the PRS estimation in the Caribbean Hispanic cohort are included in the following sections.

### PRS estimation in the NIA‐LOAD FBS families using LDPred2

To establish the robustness of the PRS computed using PRSice, we computed PRS using LDPred2,^23^ a Bayesian method that infers the posterior mean effect size of each marker by using a prior on effect sizes and LD information from an external reference panel. For input, we used genome‐wide SNPs excluding the 2 MB region flanking the *APOE* region. LD was estimated with Europeans from the 1000Genomes panel as well as the NIA‐LOAD dataset (the PRS was nearly identical using either reference panel). We use the LDPred2‐auto option which automatically estimates the *p*‐value and the SNP heritability. This avoids the need for validation data to tune hyper‐parameters. For PRS estimates obtained using LDPred2, we computed association with AD status and compared the penetrance and recurrence risk estimates with PRS obtained from PRSice. In addition, we checked the correlation of the PRS computed using the two methods, particularly in the highest and lowest quintiles. It has been observed that these PRSice and LDPred2 provide the best polygenic prediction.^24^

### PRS estimation in Caribbean Hispanic cohort

There were no large, published genome wide array studies of Alzheimer's Disease in Caribbean Hispanics. Therefore, we split the cohort of Caribbean Hispanics into two sets,^12^ (a) the training sample (60% of the dataset) to perform a GWAS and obtain summary statistics for association genome‐wide SNPs and (b) a test sample to compute the PRS and to compute the penetrance and recurrence risk in families (40% of the dataset). GWAS in the training sample was performed using generalized mixed model with Alzheimer's Disease as an outcome and first three principal components, sex, age, and genetic relationship matrix (GRM) as fixed and random effects, respectively. GRM was constructed using the GEMMA^25^ software (v0.98). GWAS analyses were conducted employing the GMMAT^26^ (v1.1) R package. After obtaining genome‐wide summary statistics, PRS was computed as described above. SNPs with a MAF ≤0.01 were included in the PRS calculation and clumped for LD (SNPs were clumped in 250 kb blocks, *p*‐value threshold = 1, clumping *r* ^2^ < 0.2). The first three PCs were included as covariates in the calculation of the PRS.

### Creating and analyzing sibships

Penetrance and recurrence risk was computed primarily in sibships because the number of living parents were limited. We conducted each analysis using two different strategies: (a) first, we chose one sibling pair from each family randomly to construct a dataset and repeated the process 100 times, (b) second, we constructed a dataset representing the maximum number of sibling pairs in each family using a family member only once (four siblings will result in two pairs, five will result in two pairs and one sibling left out and so on). We repeated this process 100 times to create replicate datasets where all family members are represented at least once. We computed penetrance and recurrence risk independently in each of the dataset and report the mean and standard deviation of the estimates.

### Computation of penetrance

The empirical penetrance of *APOE‐ε4* was computed and followed by dividing the PRS distribution into quintiles to determine the proportion or penetrance of Alzheimer's disease among individuals within each quintile and decile of the distribution. We computed penetrance in the full dataset and subsequently restricted the analyses to siblings because of the small number of living parents with a late onset disease. We computed penetrance (and recurrence risk) in sibships using two strategies: (a) one sibling pair per family, (b) all sibling pairs in a family (see above). Next, we estimated the penetrance of PRS in discordant sib‐pairs in which one sibling had Alzheimer's disease and the other sibling was unaffected. Within discordant sib‐pairs penetrance of PRS was calculated by computing the proportion of sibships where the affected sibling had the higher PRS.

### Age specific cumulative penetrance of Alzheimer's disease

The penetrance calculations described above did not account for age of the siblings. We used a nonparametric, genotype‐specific risk estimation method allowing covariates^27^ to compute age‐specific cumulative penetrance or lifetime risk of Alzheimer's disease in all family members, adjusted for sex and principal components representing population substructure. We coded individuals in the highest quintile of the PRS distribution as carriers and considered the individuals in the lowest quintile as non‐carriers. Statistical significance between risk estimates at different age groups (for e.g., *APOE‐ε4* carriers and non‐carriers and PRS highest quintile carriers versus lowest quantile carriers) was computed after 1000 permutations of the case–control designation, which allowed derivation of risk estimates and determination of statistical significance of the true estimate.

### Recurrence risk calculation

A liability threshold model^28^ was used to compute recurrence risk of *PRS* at different quintiles of the distribution using (a) disease prevalence, (b) PRS quintile threshold and (c) the amount of genetic variance explained by the PRS as parameters. In addition to recurrence risk in the highest quintile, we also computed recurrence risk at highest 1st, 5th, and 10th percentiles. Within sibships, we computed the probability of Alzheimer's disease recurrence as proportion of younger siblings affected when the older affected sibling was in the highest quintile PRS. We computed recurrence risk using one sibling‐pair per family and using all pairs in a family.

## Results

### Polygenic risk score in Alzheimer's disease

**Figure 1:**
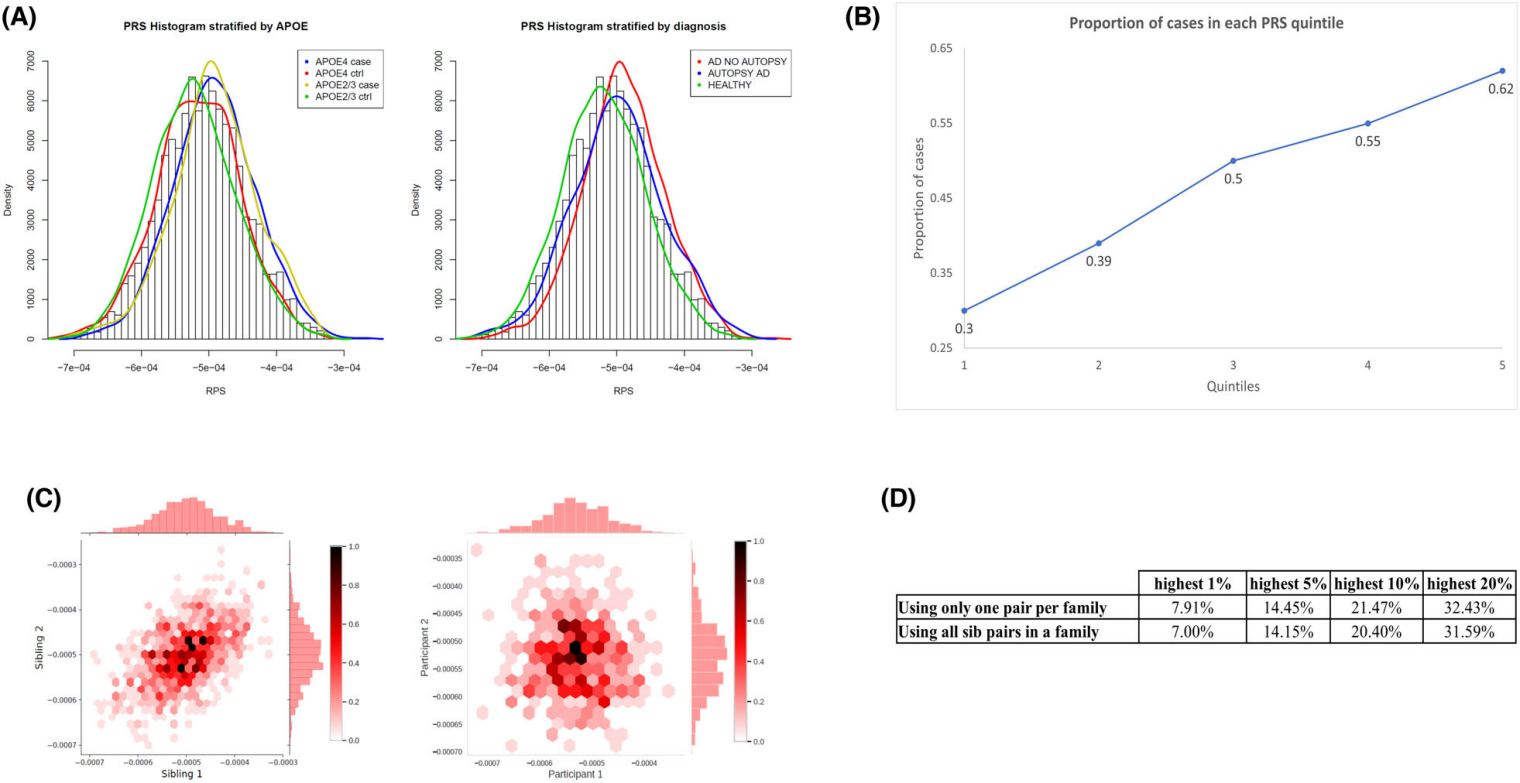
PRS in the NIALOAD dataset. (A) Distribution of PRS by AD and *APOE‐ε4* status in NIALOAD cohort (left) and distribution of PRS in clinical and autopsy confirmed AD and healthy controls (right). (B) Proportion of cases by each quintile of the PRS (NIA‐LOAD). (C) Correlation of PRS in siblings and unrelated pairs of individuals. One sibling pair was randomly selected from each family (666 families) to construct a dataset. This process was repeated 100 times. The paired correlation between the PRS of siblings and *p*‐value were calculated for each dataset. The average sibling correlation of PRS in the 100 iterations was 0.52. Among 846 unrelated individuals, were created random pairs and computed the PRS correlation for comparison. The left panel shows sibling correlation of PRS for one instance (correlation = 0.50 and *p* = 6.1e‐60). The right panel shows the PRS correlation among pairs of unrelated individuals −0.06 (*p* = 0.23). (D) Percentage of siblings with PRS in the highest 1st, 5th, 10th and 20th percentile when the other sibling also has PRS within that threshold.

The PRS, calculated in 3676 participants (Table 1) comprised of 50,637 SNPs genome‐wide (excluding the *APOE* region) that were associated with clinical diagnosis of AD at a nominal *p*‐value <0.1. PRS was robustly associated with the clinical diagnosis of Alzheimer's disease (odds ratio = 1.6 [1.51–1.73], *p* < 10e‐16) (Fig. 1‐I, Table 2). Within the highest quintile of the PRS distribution, 62% of the individuals were affected compared to 24.58% affected in the lowest quintile (Fig. 1‐II). Among the family members, 352 individuals had autopsy‐confirmed Alzheimer's disease, and the mean PRS in those individuals was significantly higher than in controls (OR = 1.43 [1.26–1.63], *p* = 7.7e‐08) (Fig. 1‐I). Compared with *APOE ε4 carriers* (1.53 [1.4–1.68]), *APOE ε4* non‐carriers had a slightly higher odds ratio for the highest PRS quintile (1.74 [1.53–1.91]). By adding the effect sizes of *APOE ε2* and *APOE‐ε4* SNPs to PRS (PRS.AD), we further strengthened the association of PRS.AD (OR = 2.1 [1.92–2.25]) in comparison to PRS excluding the *APOE* region.

**Table 2:** PRS association with Alzheimer's disease, amount of phenotypic variability explained, and recurrence risk conferred by PRS.

MODEL	NIA‐LOAD					
	OR (of the PRS)	*R* ^2^	Recurrence risk			
			99th percentile	95th percentile	90th percentile	80th percentile
AD ~ PRS^1^ + AGE + SEX + PCs	1.6 (1.51–1.73)	0.068	0.22	0.19	0.18	0.17
AD ~ PRS.AD^2^ + AGE + SEX + PCs	2.1 (1.92–2.25)	0.12	0.25	0.22	0.2	0.19
AD ~ PRS^1^ + AGE + SEX + PCs within *APOE ε4* carriers	1.53 (1.4–1.68)	0.093	0.23	0.21	0.19	0.18
AD ~ PRS^1^ + AGE + SEX + PCs within *APOE ε4* non‐carriers	1.74 (1.53–1.91)	0.074	0.22	0.2	0.19	0.17

MODEL^3^	EFIGA					
	OR (of the PRS)	*R* ^2^	Recurrence risk			
			99th percentile	95th percentile	90th percentile	80th percentile
AD ~ PRS^1^ + AGE + SEX	1.425 [1.31–1.53]	0.036	0.19	0.18	0.17	0.16
AD ~ PRS.AD^2^ + AGE + SEX	1.433 [1.31–1.56]	0.036	0.19	0.18	0.17	0.16
AD ~ PRS^1^ + AGE + SEX + APOE4	1.38 [1.27–1.5]	0.066	0.21	0.19	0.18	0.17

^1^ PRS was computed genome‐wide but excluding variants in the 2 MB window around the APOE gene (human genome b38, chromosome 19: 42,905,791–46,909,393).

^2^ PRS.AD represents the polygenic risk score that includes the effect of APOE ε4 and ε2 alleles. PRS.AD constructed with genome‐wide SNPs excluding the APOE region, but including the effect sizes SNPs that code for APOE‐ε4 (rs429358) and ‐ε2 (rs7412) alleles. The APOE‐ε4 SNP rs429358 was not genotyped or imputed in the Caribbean Hispanic dataset, we used rs769449 as a proxy for APOE‐ε4 genotype. The Pearson correlation between number of APOE‐ε4 alleles and rs769449 is 0.56 (*p* < 1e‐16).

^3^ The first three principal components (PCs) in the EFIGA families were significantly associated with clinical diagnosis of Alzheimer's Disease. The PRS was calculated by adjusting for PCs as covariates. To determine the amount of recurrence risk conferred by the PRS alone, we excluded PCs from the association analyses (and used the R^2^ estimates of the regression model to compute recurrence risk).

Using LDPred2 to compute the PRS independently in the cohort, we observed a similar robust association of the PRS with Alzheimer's disease (OR = 1.57 [1.46–1.68], *p* < 1e‐16). Overall correlation between the PRS computed using PRSice and LDpred2 was moderate (0.38 *p* = 4.53e‐153) and 43% of individuals in the highest PRS quintile calculated by PRSice were in the highest quintile of LDPred2. As previously noted,^5^ different PRS methods have similar prediction accuracies assigning different scores to individuals, but these are considered among the best methods.^24^ However, because individuals at the extremes of the PRS distribution were highly concordant using the two different methods, we used the results from PRSice for the rest of the analyses.

In patients with Alzheimer's disease, the PRS was associated with a lower age at onset (1.4 years difference between mean at age at onset between the top and bottom quintiles of the PRS, *p* = 0.002) (Table 3). Among *APOE‐ε4* non‐carriers, patients with Alzheimer's disease in the highest PRS quintile had 4.1 years younger age of onset of disease (*p* = 0.0002) compared to patients in bottom quintiles, but only 0.43 years in *APOE‐ε4* carriers.

**Table 3:** Association of age at Onset of AD in patients with PRS.

SAMPLE	MODEL	Effect size on age at onset (in years)	*p*‐value (of the PRS)	Mean age at onset in highest quintile PRS	Mean age at onset in lowest quintile PRS
All NIALOAD cases	AAO ~ APOE4num + PCs	−3.44	<2e‐16		
All EFIGA cases		−2.92	7.70E‐15		
All NIALOAD cases	AAO ~ PRS + PCs	−0.61	0.0017	74.12	75.5
NIALOAD cases with at least one *APOE ε4* allele		−0.3	0.17	73.09	73.53
NIALOAD cases with no *APOE ε4* allele		−1.36	0.00024	76.44	80.5
All EFIGA cases		−1.15	6.10E‐08	74.5	77.91
EFIGA cases with at least one *APOE ε4* allele		−0.94	0.0018	73.31	75.8
EFIGA cases with no *APOE ε4* allele		−1.1	0.00018	75.75	79.34

### PRS concordance by relatedness

As expected, the correlation of PRS among sib‐pairs was 0.52 (*p* < 10e‐60) (Fig. 1‐III), compared to minimal correlation in randomly draws pairs from 846 unrelated individuals (R = ‐0.06, *p* = 0.22). We computed the frequency that a sibling had a PRS within the same distribution as their co‐sibling in highest 1^st^, 5^th^, and 10^th^ percentiles or in the highest quintile of the PRS distribution (Fig. 1‐IV).^29^ Among siblings in the highest 1% PRS distribution, 7.91% of co‐siblings also had a PRS in the top 1%, corresponding to an eight‐fold enrichment. Among siblings whose PRS was within the highest quintile, 32.43% of their co‐siblings also had a PRS in the top quintile.

### Penetrance of PRS

Estimates of penetrance in the highest PRS quintile (0.62) was equal to the penetrance of genotypes containing at least one *APOE‐ε4* allele (0.62) and both were substantially higher than penetrance of *APOE‐ε4* non‐carriers (0.3) and the lowest quintile PRS (0.3) (Table 4). Compared with *APOE‐ε4* carriers in the lowest quintile of PRS distribution (0.48), the penetrance of *APOE‐ε4* carriers within the highest quintile PRS increased to 0.74. Within non‐carriers of *APOE*‐*ε4*, penetrance in the highest quintile PRS was also increased compared to the lowest quintile (0.44 vs. 0.16). Restricting to siblings only within families, the penetrance within the highest PRS quintile (0.74) was slightly higher than the penetrance of *APOE‐ε4* allele (0.71) and increased to 0.77 within the highest PRS decile.

**Table 4:** Penetrance of PRS and *APOE‐ε4* allele in the NIA‐LOAD and EFIGA cohorts.

	Penetrance			
	In everyone		In sibships	
	APOE alone	PRS^1^	APOE alone	PRS^1^
*NIALOAD*				
*APOE ε4* allele	0.62		0.71	
Non‐*APOE ε4* allele (*APOE ε3* or *ε2 allele*)	0.3		0.47	
PRS in highest quintile		0.62		0.74
PRS in lowest quintile		0.3		0.48
*APOE ε4* carriers and PRS in the highest quintile		0.74		0.81
*APOE ε4* carriers and PRS in the lowest quintile		0.48		0.57
*APOE ε4* non‐carriers and PRS in the highest quintile		0.44		0.62
*APOE ε4* non‐carriers and PRS in the lowest quintile		0.16		0.31
PRS in highest decile		0.64		0.77
PRS in lowest decile		0.27		0.46
*APOE ε4* carriers and PRS in the highest decile		0.77		0.84
*APOE ε4* carriers and PRS in the lowest decile		0.43		0.57
*APOE ε4* non‐carriers and PRS in the highest decile		0.47		0.64
*APOE ε4* non‐carriers and PRS in the lowest decile		0.15		0.28
*EFIGA*				
*APOE* e4 carriers	0.6		0.63	
*APOE* e4 non‐carriers	0.44		0.48	
Highest quintile		0.6		0.63
Lowest quintile		0.48		0.53
*APOE* e4 carriers and highest quintile		0.703		0.67
*APOE* e4 carriers and lowest quintile		0.53		0.52
*APOE* e4 non‐carriers and highest quintile		0.53		0.58
*APOE* e4 non‐carriers and lowest quintile		0.45		0.54

^1^ Excludes single nucleotide polymorphisms in the 2 MB region surrounding APOE.

We found that in 61.2% (±2%) of all discordant sibships (Table 5), the sibling with the higher PRS was affected. Restricted to participants in the highest PRS quintile, the PRS predicted Alzheimer's disease correctly 82% of the time in discordant sibling pairs. Within discordant sibships where both siblings were *APOE‐ε4* carriers, the highest PRS quintile predicted Alzheimer's disease 85% of the time compared to 35% in the lowest quintile. Within *APOE‐ε4* non‐carriers, the highest PRS quintile predicted the Alzheimer's disease 72% of the time compared to 14% for the lowest quintile.

**Table 5:** Penetrance of PRS in discordant sibling pairs in the NIA‐LOAD cohort.

	Proportion of discordant sibships with the higher PRS sibling being affected^1^	
	One sibling pair per family	Maximal sibling pairs per family
Overall	0.61 (0.02)	0.60 (0.01)
PRS in highest quintile	0.82 (0.03)	0.82 (0.02)
PRS in Lowest quintile	0.31 (0.06)	0.31 (0.05)
*APOE ε4* carriers and highest quintile	0.85 (0.04)	0.86 (0.03)
*APOE ε4* carriers and lowest quintile	0.35 (0.10)	0.35 (0.07)
*APOE ε4* non‐carriers and highest quintile	0.72 (0.06)	0.72 (0.05)
*APOE ε4* non‐carriers and lowest quintile	0.14 (0.13)	0.14 (0.10)
PRS in highest decile	0.88 (0.05)	0.88 (0.04)
PRS in lowest decile	0.26 (0.07)	0.24 (0.05)
*APOE ε4* carriers and highest decile	0.85 (0.06)	0.88 (0.05)
*APOE ε4* carriers and lowest decile	0.32 (0.11)	0.31 (0.08)
*APOE ε4* non‐carriers and highest decile	0.89 (0.10)	0.88 (0.09)
*APOE ε4* non‐carriers and lowest decile	0.09 (0.13)	0.11 (0.97)

^1^ From each family, one discordant sibship was randomly chosen to create a dataset. Proportion of discordant sibships where the sibling with the higher PRS was affected was computed. This process was repeated 100 times and the mean (standard deviation) of the proportion is reported.

### Age‐specific cumulative penetrance of PRS and *APOE* genotype

**Figure 2:**
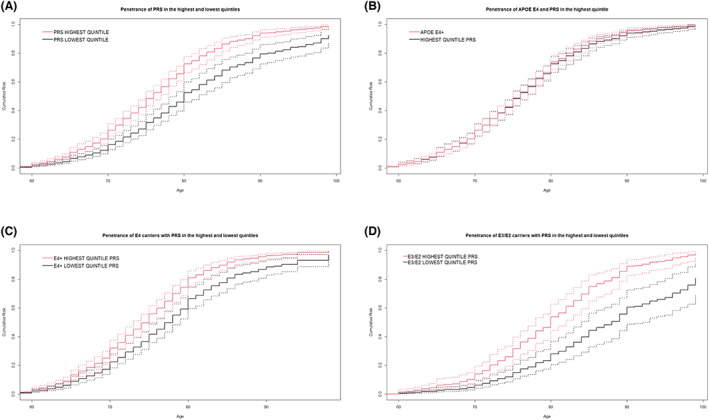
Age specific cumulative penetrance comparisons between various groups. The solid lines represent the point estimates of penetrance from 1000 iterations and the dotted lines show the 95% confidence intervals. (A) Age‐specific cumulative penetrance of highest quintile and lowest quintiles PRS. (B) Age‐specific cumulative penetrance in highest quintile PRS versus *APOE‐ε4* allele. (C) Age specific cumulative penetrance in highest and lowest quintile PRS among *APOE‐ε4* carriers. (D) Age specific cumulative penetrance of highest quintile and lowest quintile PRS among *APOE ε4* non‐carriers (*APOE ε3 or ε2* carriers).

The age‐specific cumulative penetrance, of Alzheimer's disease was computed in families for (a) *APOE‐ε4* carriers, (b) *APOE‐ε4* non‐carriers, (c) the highest and lowest PRS quintiles, (d) the highest and lowest PRS quintile among *APOE‐ε4* allele carriers and (e) highest and lowest PRS quintiles among *APOE‐ε4* non‐carriers. The penetrance of the highest quintile PRS (Fig. 2A) (26.3% [22.3–30.8%]) was significantly higher (*p* = 0.0006) than the lowest quintile PRS at age 70 (16.2% [12.8–20%]). At age 85, the penetrance increased to 86.3% [81.4–90.3%] in the highest quintile and 68% [59.5–75.4%] in the lowest quintile (*p* = 0.0001). The age‐specific penetrance of having an *APOE‐ε4* allele was not significantly different from being in the highest PRS quintile (Fig. 2B). The penetrance of Alzheimer's disease with an *APOE‐ε4* allele increased from 10.9% [8.2–14%] at age 65 to 88.4% [83.7–91.9%] at age 85. Identical penetrance was observed for carriers in the highest PRS quintile (Fig. 2B). Compared to *APOE‐ε4*, at age 65, the penetrance of highest PRS quintile was 11% [7.9–14.2%] (*p* = 0.99), increasing to 86.3% [81.4–90.3%] (*p* = 0.5) at age 85.

*APOE‐ε4* carriers who were in the highest PRS quintile also had a significantly higher penetrance of Alzheimer's disease than *APOE‐ε4* carriers in the lowest PRS quintile from ages 70 to 85 (Fig. 2C). At age 70, *APOE‐ε4* carriers within the highest PRS quintile PRS had a penetrance of 32% [27–37.6%] compared to 22.4% [18.3–27.9%] for lowest quintile (*p* = 0.011). At age 85, the penetrance for highest PRS quintile increased to 92% [88.4–94.7%], compared to 80.8% [73.3–87.6%] for the lowest quintile (*p* = 0.0034). For *APOE‐ε4* carriers, being in the highest PRS quintile represented an 9.5–11.1% higher risk at ages 70 and 85 respectively, with the maximum difference in risk at age 80 (14.5%).

Similarly, *APOE‐ε4* non‐carriers in highest quintile of the PRS had a significantly higher probability of Alzheimer's disease than those in the lowest PRS quintile between ages 70 and 85 (difference of 6.7% at age 70 to 30.5% by age 85) (Fig. 2D). At age 70, *APOE‐ε4* non‐carriers with highest quintile PRS had a penetrance of 13.93% [9.6–19.5%] compared to 6.23% [4–9.25%] for lowest quintile PRS carriers (*p* = 0.008). At age 85, the penetrance for highest quintile PRS increases to 74.94% [64.5–82.9%], compared to 44.3% [32.9–56.25%] for the lowest quintile (*p* = 0.0001).

### Recurrence risk of disease in siblings

We used the estimated prevalence of Alzheimer's disease for individuals over age 65 years of 13%^30^ as a reference to determine the influence of the PRS and *APOE* genotype on recurrence risk. The proportion of variance explained by the PRS alone and by PRS with *APOE* alleles was 0.068 and 0.12 respectively (Table 2). We estimated the recurrence risk for siblings at the highest PRS quintile, the highest PRS decile and the top 5^th^ and 1^st^ percentiles (Table 6). For individuals in the highest PRS quintile, the recurrence risk of Alzheimer's disease was 17%, but it increased to 19% when including the *APOE* alleles in the PRS score. While the lifetime risk of developing Alzheimer's disease in the general population is 10–12%,^31^, ^32^ first‐degree relatives have a significantly higher recurrence risk of 20–25%. This indicates that affected siblings in the highest PRS quintile confer approximately 70% additional lifetime risk of Alzheimer's disease to their co‐sibling and together with the *APOE* alleles explains nearly half of the recurrence risk.

**Table 6:** Recurrence risk in NIA‐LOAD: Recurrence risk of a sibling with an affected proband carrying a high PRS (at different percentile levels of the distribution).

Age‐range	Age specific prevalence of Alzheimer's disease (%)	Variable	Recurrence risk at PRS percentile level			
			99th percentile	95th percentile	90th percentile	80th percentile
65–74 years	3	PRS^1^	0.06	0.05	0.05	0.04
		PRS.AD^2^	0.08	0.06	0.06	0.05
75–84 years	17	PRS	0.27	0.24	0.23	0.22
		PRS.AD	0.31	0.27	0.26	0.24
85 and above years	32	PRS	0.45	0.42	0.40	0.39
		PRS.AD	0.50	0.46	0.43	0.41

^1^ PRS was computed genome‐wide but excluding variants in the 2 MB window around the APOE gene (human genome b38, chromosome 19: 42,905,791–46,909,393).

^2^ PRS.AD represents the polygenic risk score that includes the effect of *APOE ε4* and *ε2* alleles. PRS.AD constructed with genome‐wide SNPs excluding the APOE region, but including the effect sizes SNPs that code for *APOE‐ε4* (rs429358) and ‐*ε2* (rs7412) alleles.

Both lifetime and recurrence risk of Alzheimer's disease are dependent on the ages of the proband and siblings. We used the age specific prevalence estimates of Alzheimer's disease based on predictions from the 2010 census^33^ to calculate the age‐specific recurrence risk conferred by a proband with a PRS at different levels of the distribution (Table 6). A person in the highest PRS quintile confers 4.0%, 22.0% and 39.0% recurrence risk to family members at ages 65–74, 74–80 and 85 and above respectively. Reinterpreted, PRS confers 33%, 29.4% and 21.9% additional risk at each age grouping 65–74, 74–80 and 85 and above.

We subsequently calculated the empirical probability of an individual being affected when their sibling is affected and in the highest PRS quintile. When a sibling was affected and in the highest PRS quintile, the proportion of affected co‐siblings was 77.24% (±3.76) (Table 7). Similarly, 78.09% (±3.25%) siblings of *APOE‐ε4* patients and 75% (±2.3%) siblings of *APOE‐ε4* non‐carrier patients were also affected.

**Table 7:** Recurrence risk in NIA‐LOAD: Empirical recurrence risk in siblings when probands are affected and carry a risk genotype (*APOE* or PRS in the highest quintile).

	Empirical recurrence risk^1^			
	maximum sib‐pairs per family		One sib pair per family	
	*APOE*	PRS	*APOE*	PRS
Proportion of younger sibling affected when older sibling is affected and is a *APOE ε4* carrier	0.78 (0.03)		0.80 (0.03)	
Proportion of younger sibling affected when older sibling is affected and is a *APOE ε4* non‐carrier)	0.75 (0.02)		0.79 (0.03)	
Proportion of younger sibling affected when older sibling is affected and has PRS in the highest quintile		0.77 (0.04)		0.78 (0.05)

^1^ From each family, one sibship was randomly chosen to create a dataset. Empirical recurrence risk was computed as the proportion of sib‐pairs where the younger sibling was affected when the older sibling affected and carried a *APOE ε4* allele (or had a highest quintile PRS). This process was repeated 100 times and the mean (standard deviation) of the proportion is reported. Similarly, for each family, maximum sib‐pair dataset was formed using each sibling only once (for example, 3 siblings can contribute one pair, 4 will contribute two pairs and so on). The empirical recurrence risk was re‐computed and the mean (standard deviation) across 100 iterations are reported.

The NIA‐LOAD FBS contributed 0.05% of the sample size used to compute effect‐size estimates for SNPs in the meta‐analysis study.^22^ Thus, the PRS in in this group could be slightly inflated.^34^ We computed PRS within the full dataset and separately in a non‐overlapping but smaller group of families that had been recruited later and observed nearly identical values. We also observed similar penetrance and recurrence risk values in the non‐overlapping groups. In addition, we compared the penetrance of PRS and recurrence risk within *APOE‐ε4* strata which minimizes the risk of over‐estimation of PRS in overlapping samples.

### Polygenic risk score in Caribbean Hispanic families

Genome‐wide effect‐size estimates were obtained by testing association in 5110 individuals (Table 1). The *APOE* locus was the only genome‐wide significant association observed in the dataset and the type‐1 errors were well controlled. The PRS in Caribbean Hispanics computed using PRSice was robustly associated (OR = 1.425 [1.31–1.55], *p* = 2.2e‐16) with Alzheimer's disease (Table 2), although the effect size of association was slightly smaller than the NIA‐LOAD FBS. The amount of genetic variance explained by the PRS excluding *APOE‐ε4* (0.036) and then including *APOE‐ε4* (0.065) was also observed to be smaller than NIA‐LOAD FBS. PRS computed with LDPred2 with highly concordant with estimates from PRSice (correlation = 0.95). Higher PRS was associated with lower age at onset (Table 3) in cases (*p* = 6.10E‐08, 3.4 years lower age of onset in the highest quintile compared to the lowest quintile). Similar to the NIA‐LOAD families, PRS had a larger effect of *APOE‐ε4* non‐carriers (*p* = 1.8e‐4, 3.59 years lower onset age in the highest quintile) compared to *APOE‐ε4* carriers (*p* = 1.8e‐03, 2.49 years lower onset age in the highest quintile). The penetrance of the highest PRS quintile (0.6) was not statistically different from penetrance of genotypes containing *APOE‐ε4* (0.6) (Table 3). However, penetrance estimates including *APOE‐ε4* non‐carriers (0.44) were observed to be higher than in NIA‐LOAD FBS. Compared with *APOE‐ε4* carriers in the lowest quintile of PRS distribution (0.53), the penetrance of *APOE‐ε4* carriers within the highest quintile PRS was increased (0.7). Within *APOE‐ε4* non‐carriers, penetrance in the highest quintile PRS was also significantly increased compared to the lowest quintile (0.53 vs. 0.45). The recurrence risk for individuals in the highest PRS (excluding *APOE‐ε4* allele) quintile of disease was 16% compared to 17% when including the *APOE‐ε4* allele.

## Discussion

In this investigation we found that a PRS above or below a certain threshold allows estimation of penetrance and recurrence risk for family members regardless of their *APOE‐ε4* carrier status. As previously observed, *APOE* explains a significant proportion of the known genetic heritability in Alzheimer's disease while the remainder the genome PRS explains only about 7–10%. However, we found the overall penetrance of Alzheimer's disease in the highest quintile of the PRS to be identical to the penetrance of the *APOE‐ε4* alone. Similarly, cumulative lifetime penetrance of disease in the highest PRS quintile (includes high‐risk *APOE‐ε4* carriers and non‐carriers) was identical to *APOE‐ε4* carriers at different ages. Within sibships, the highest quintile of the PRS had a higher proportion of affected individuals than *APOE ε4* carriers with a higher penetrance observed in the top decile of the PRS distribution. More importantly, within the *APOE‐ε4* carrier group and the non‐carrier group, PRS stratifies high and low risk individuals into top and bottom quintiles with significant differences in age‐specific penetrance. Because the highest PRS quintile consists of individuals with and without *APOE ε4*, this approach can potentially identify, *APOE‐ε4* non‐carriers with similar risk profiles as high‐risk ε4 carriers. Individuals within the highest PRS quintile had a statistically significant higher overall and age‐dependent risk regardless of their *APOE* genotype. Differences in penetrance and lifetime risk between highest and lowest PRS quintiles was most pronounced among individuals within *APOE‐ε4* non‐carriers.

We observed the recurrence risk for siblings whose co‐sibling was affected and in the highest PRS quintile was increased by 70–90%. Previous estimates suggested that siblings of patients with Alzheimer's disease have 25–30%^31^ increased risk of disease, and we found that most of this excess risk was captured by being in the highest quintile of the PRS supporting our contention that the PRS can be used to identify individuals at high risk.

These observations have clear implications for families with late onset Alzheimer's disease. The *APOE‐ε4* allele is highly penetrant and associated with elevated recurrence risk, but present in only 15% of the white, non‐Hispanic population. *APOE* allele frequency varies considerably in other ethnic groups.^35^ The absence of *APOE‐ε4* does not ensure protection from Alzheimer's disease. Thus, the discovery of additional risk loci favors the use of the PRS to provide comprehensive estimates of penetrance and recurrence risk in families affected by Alzheimer's disease. The highest quintile of the PRS distribution has similar penetrance and recurrence risk as *APOE‐ε4* alone. However, the findings here indicate that after determining the presence or absence of *APOE ε4*, the PRS can be further used to stratify high and low risk ε4 carriers and identify at‐risk individuals that do not carry ε4 allele. Risk assessment using APOE genotype and establishing PRS thresholds can identify at‐risk individuals that might benefit from frequent monitoring and preventative interventions. In particular, PRS can be valuable in APOE ε4 non‐carriers for risk assessment and stratification. There may be some limitations to our findings. The PRS was applied to families selected because they had affected family members who were able to be genotyped. The motivation to participate may inflate the effects reported here on penetrance and recurrence risk of the PRS and *APOE‐ε4*. We believe that the NIA‐LOAD FBS families are representative of the general population. The Caribbean Hispanic families represent a slightly inbred, admixed set of families. The PRS was equally effective in assessing penetrance and recurrence risk in these families. The development of the PRS requires the incorporation of effect sizes for common variants throughout the genome from ethnic groups of similar ancestry. Widespread commercially available genotyping could be used to develop ethnic‐specific PRS for clinical use, but it would require analyses of individual genome‐wide studies of common variants to provide accurate effect sizes.

The ongoing genetic investigations will increase the number of genes to consider, understanding how each gene causes disease will be difficult because the contribution of each variant may be small. Using PRS to focus on genes of similar function may identify variants that fit into specific pathways suggesting a potential target for therapeutic investigation. The study here indicates PRS is a powerful clinical tool to provide high‐risk families with information concerning their individual risk for Alzheimer's disease.

## Author Contributions

Richard Mayeux and Badri N. Vardarajan conception and design of the study. Dolly Reyes‐Dumeyer, Kelley Faber, Alison Goate, Carlos Cruchaga, Brad Boeve, Margaret Pericak‐Vance, Jonathan L. Haines, Roger Rosenberg, Debby Tsuang, Robert A. Sweet, David A. Bennett, Tatiana Foroud and Richard Mayeux data acquisition. Min Qiao, Annie J. Lee, Michael Chao, Alan Renton, Badri N. Vardarajan conducted statistical analyses. Annie J. Lee, Giuseppe Tosto, Carlos Cruchaga, Margaret Pericak‐Vance, Jonathan L. Haines, Roger Rosenberg, Debby Tsuang, Robert A. Sweet, David A. Bennett, Robert S. Wilson, Tatiana Foroud, Richard Mayeux, and Badri N. Vardarajan drafted and edited the manuscript.

## Conflict of Interest

Each co‐author's conflict of interest is listed below. M.Q, A.J.L, D.R.D, K.F and M.C. do not have any conflicts of interest. G.T., C.C., M.P.V, and R.S.W have grants from the NIH. A.G. has grants from the NIA/NIH and receives money from Athena Diagnostics for licensing of TDP43 mutations, and has consulted for UK Dementia Research Institute, UK VIB, Katholik University, Leuven, Belgium Center for Molecular Neurology, Antwerp, Belgium Queensland Brain Institute, Brisbane, Australia. A.R. has grants from the NIA/NIH, the Alzheimer's Association, and from the JPB Foundation. B.B. has grants from the NIH, receives royalties as a co‐editor of a textbook on dementia, and is on the Scientific Advisory Board (SAB) for the Tau Consortium. J.H.L has received consulting fee from University of Miami and University of Miami. He holds grants from NIH. R.R. has grants from the NIA/NIH and The Zale Foundation and receives license/royalty fees from Elsevier Publishing Inc., Springer Publishing Inc.; payments from Elsevier, Springer and Vitruvian, Inc., and The American Academy of Neurology; and he has a 2009 patent on an Amyloid Beta Gene vaccine. D.T. has grants from the NIA/NIH and receives consulting fees from Acadia Pharma and is on the SAB for the Lewy Body Association. R.A.S has grants from the NIA/NIH and National Institute of Mental Health of the NIH. D.A.B. is a part of AbbVie's data monitoring board, a consultant with Takeda Inc, Origent Inc and SBIR. He consults with Vigorous Minds (unpaid). He has received NIH funding is a member of professional societies including the National Academy of Sciences and has given grand rounds talks. T.F. has grants from the NIA/NIH, The Department of Defense, and the Michael J. Fox Foundation; is on the SAB for several academic institutions; and receives support from Northwestern University for Continuing Medical Education. R.M. has grants from the NIA/NIH and is on the SAB for the Rush Alzheimer's Disease Research Center. B.N.V has grants from the National Institute on Aging (NIA) of the National Institutes of Health and the Department of Defense and is a cancer bioinformatics consultant for Kodikaz Therapeutics.
